# Machine learning algorithms reveal the secrets of mitochondrial dynamics

**DOI:** 10.15252/emmm.202114316

**Published:** 2021-05-27

**Authors:** Jack J Collier, Robert W Taylor

**Affiliations:** ^1^ Wellcome Centre for Mitochondrial Research, Translational and Clinical Research Institute Faculty of Medical Sciences Newcastle University Newcastle upon Tyne UK; ^2^ NHS Highly Specialised Service for Rare Mitochondrial Disorders Newcastle upon Tyne Hospitals NHS Foundation Trust Newcastle upon Tyne UK

**Keywords:** Genetics, Gene Therapy & Genetic Disease, Neuroscience

## Abstract

Mitochondria exist as dynamic networks whose morphology is driven by the complex interplay between fission and fusion events. Failure to modulate these processes can be detrimental to human health as evidenced by dominantly inherited, pathogenic variants in *OPA1*, an effector enzyme of mitochondrial fusion, that lead to network fragmentation, cristae dysmorphology and impaired oxidative respiration, manifesting typically as isolated optic atrophy. However, a significant number of patients develop more severe, systemic phenotypes, although no genetic modifiers of OPA1‐related disease have been identified to date. In this issue of *EMBO Molecular Medicine*, supervised machine learning algorithms underlie a novel tool that enables automated, high throughput and unbiased screening of changes in mitochondrial morphology measured using confocal microscopy. By coupling this approach with a bespoke siRNA library targeting the entire mitochondrial proteome, the work described by Cretin and colleagues yielded significant insight into mitochondrial biology, discovering 91 candidate genes whose endogenous depletion can remedy impaired mitochondrial dynamics caused by OPA1 deficiency.

## Graphical abstract

J. Collier and R. Taylor discuss a high‐throughput screening approach to identify suppressors of mitochondrial fragmentation in OPA1 patient fibroblasts as reported by Cretin et al, in this issue of *EMBO Mol Med*.

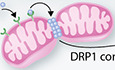

Margaret Reed Lewis’ seminal contributions to cell biology influenced research across the field. Not only did Lewis pioneer tissue culture techniques, but working with her husband, Warren Lewis, she was among the first scientists to observe mitochondrial dynamics, meticulously illustrating fission and fusion events in chick heart tissue (Lewis & Lewis, 1915). Their work clearly demonstrated that mitochondria manifest in many different shapes, from punctate structures to networks that spread throughout the cytoplasm (Fig 1A). The century proceeding this work provided a body of evidence that has demonstrated that the balance of fission and fusion modulates mitochondrial morphology, supporting the cellular metabolic response to changing physiological conditions (Sprenger & Langer, 2019; Giacomello *et al,* 2020). Mitochondrial fusion increases ATP production and enables the exchange of metabolites and mitochondrial DNA (mtDNA), whereas fission stimulates ROS production and can support the autophagic degradation of mitochondria *via* mitophagy. Beyond supporting metabolic demand, reshaping the mitochondrial network contributes to stemness, differentiation, apoptosis and senescence. The identification of key drivers of mitochondrial dynamics has been critical for understanding these biological implications.

**Figure 1:**
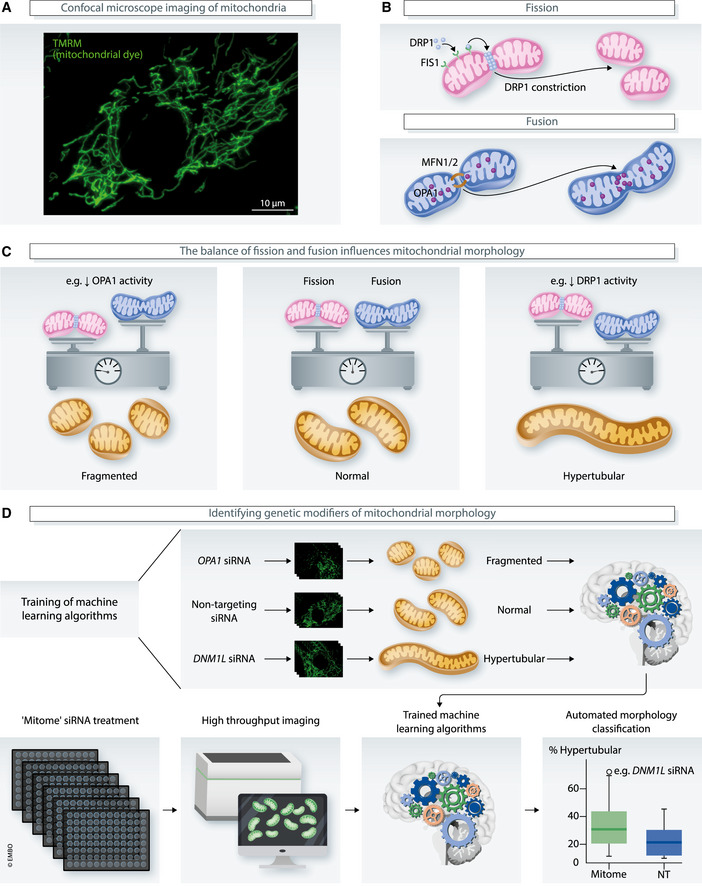
Novel approaches to investigating mitochondrial morphology (A) Confocal microscopy is routinely used to assess mitochondrial morphology, which is determined by the balance of fission and fusion events. (B) Mitochondrial fission is facilitated by DRP1, which is recruited from the cytoplasm *via* outer mitochondrial membrane protein FIS1. Conversely, two mitochondria can be tethered together by MFN1 and MFN2, before OPA1 coordinates fusion of the outer and inner mitochondrial membranes. (C) Consequently, decreased OPA1 activity causes fragmentation of mitochondria, whereas decreased DRP1 activity causes hypertubular phenotypes. (D) Cretin and colleagues have developed a novel technique that aids in discovery of genetic modifiers of mitochondrial morphology. First, researchers trained machine learning algorithms to classify mitochondrial morphology using known mitochondrial phenotypes, then combined this with an siRNA screen targeting the entire mitochondrial proteome, termed the “mitome”.

In mammalian cells, mitochondrial division is facilitated by dynamin‐related protein 1 (DRP1), a cytosolic GTPase that is recruited to the outer mitochondrial membrane (OMM) *via* FIS1, an OMM receptor, before forming oligomers that constrict leading to scission (Fig 1B). Mitochondrial fusion is also mediated by GTPases; OMM proteins Mitofusin 1 and 2 (MFN1/2) tether neighbouring mitochondria together, before OPA1, an inner mitochondrial membrane (IMM) protein, coordinates OMM and IMM fusion (Fig 1B). Disrupting the balance of fission and fusion can have remarkable effects on mitochondrial morphology, leading to mitochondrial network hypertubulation (e.g. *via* diminished DRP1 activity) or fragmentation (e.g. *via* diminished OPA1 activity), either of which impedes mitochondrial function (Fig 1C). In humans, mutations affecting these important proteins are implicated in a broad range of mitochondrial pathologies, leading to neurological impairments.

The ability to identify factors, whether they be endogenous (e.g. genetic) or exogenous (e.g. drugs), that affect mitochondrial morphology relies on accurately deciphering the state of the mitochondrial network (i.e. fragmented, normal or hypertubular). Presently, mitochondria can be illuminated in cultured cells using confocal microscopy, for example using cell‐permeant dyes that sequester in active mitochondria, fluorescent‐tagged mitochondrial proteins or classical immunocytochemistry techniques (Fig 1A). After images have been captured, segmentation of the mitochondrial network enables quantification of mitochondrial features, typically length, width and aspect ratio. Several tools now exist that streamline these processes for the analysis of individual images, notably Mitochondrial Network Analysis (MiNA; Valente *et al,* 2017). Yet, the process from image collection to analysis remains relatively low throughput and is open to user bias. This contrasts the need for automated, high throughput analysis in a field where large‐scale screening is becoming increasingly common as researchers attempt to gain a deeper understanding of mitochondrial dynamics using agnostic approaches.

To overcome these limitations of conventional mitochondrial morphology analysis, Cretin and colleagues used integrated supervised machine learning algorithms to develop a pipeline that enables automated confocal imaging, segmentation and classification of mitochondrial network morphology (Fig 1D). Algorithms were trained to define morphology based on ground truths derived from the assessment of hundreds of cells with known fragmented (*OPA1* siRNA), normal (non‐targeting siRNA) or hypertubular (*DNM1L* siRNA) phenotypes, and their robustness was demonstrated by the ability of the pipeline to identify other known regulators of mitochondrial morphology (Fig 1D). This workflow was subsequently integrated with high throughput siRNA screens against the entire nuclear‐encoded mitochondrial genome, termed the *mitome* library (Fig 1D). Screening control human fibroblasts identified 167 genes whose depletion caused fragmentation or hyperfusion of the mitochondrial network. The enormous potential of this workflow to generate new understandings of mitochondrial dynamics was evidenced by the identification of previously unreported contributors to mitochondrial morphology among the 167 genes (Cretin *et al*, 2021).

The researchers then demonstrated that their novel pipelines have the potential to significantly impact our understanding of mitochondrial disease involving impaired mitochondrial dynamics. Dominant, recessive and *de novo* dominant *OPA1* variants cause neurological disorders, with recessive variants causing the most severe presentations including fatal infantile mitochondrial encephalopathy (Spiegel *et al,* 2016). Deleterious heterozygous variants cause dominant optic atrophy (DOA) through mitochondrial fragmentation, dysregulated cristae organisation and impaired oxidative phosphorylation (Zanna *et al,* 2008). These mutations largely fall in the putative GTPase domain, so haploinsufficiency represents a likely major pathomechanism. Although optic atrophy is most commonly an isolated phenotype in patients harbouring dominant variants, it is well established that approximately 20% of these patients also demonstrate multi‐system neurological presentations, including deafness, ataxia, myopathy, peripheral neuropathy and progressive external ophthalmoplegia—so‐called “DOA^+^” features, typically affecting young adults (Hudson *et al,* 2008; Yu‐Wai‐Man *et al,* 2010). Currently, it is difficult to predict clinical outcomes in individual cases. To investigate whether genetic modifiers may contribute to the presentation of DOA^+^ features, the *mitome* library and automated pipeline were applied to a DOA^+^ patient fibroblast cell line (*OPA1^S545R^*) harbouring a heterozygous p. Ser545Arg *OPA1* variant (NM_015560.2) and exhibiting mitochondrial network fragmentation. The researchers identified 91 genes whose endogenous depletion remedied the fragmented phenotype, supporting the hypothesis that genetic modifiers may contribute to clinical outcomes in patients harbouring pathogenic *OPA1* variants.

Among these genes was *PGS1*, encoding a key enzyme within the cardiolipin synthesis pathway. Cardiolipin is an inner mitochondrial membrane‐specific phospholipid that supports mitochondrial dynamics, protein import and OXPHOS activities, the regulation of cristae maintenance and apoptosis (Paradies *et al,* 2019). Cretin and co‐workers observed that *PGS1* depletion was only able to exert an anti‐fragmentation effect when OPA1 was not completely absent, supporting its role as a genetic modifier, specifically, of hypomorphic *OPA1* variants. It is important to emphasise this discovery, because haploinsufficiency is likely an important mechanism related to dominant *OPA1* pathology.

The cardiolipin synthesis pathway as a modifier of mitochondrial morphology was further implicated by the demonstration that disruption of other genes involved in this process (*Tamm41*, *Ptpmt1* and *Cls1*) also rescued the fragmented mitochondrial network phenotype in hypomorphic *OPA1* cells. Evidence suggested the accumulation of cardiolipin precursor phosphatidic acid rather than depletion of cardiolipin underpins the mechanism protecting mitochondrial morphology. In addition, *PGS1* depletion also remedied OXPHOS impairment in hypomorphic *OPA1* cells, though it could not reverse mtDNA depletion, apoptotic sensitivity or cristae dysmorphology, thus demonstrating that the physiological consequences of OPA1 dysfunction are uncoupled.

It is easy to remark that we have come a long way in the 100 or more years since Margaret Reed Lewis painstakingly illustrated, by hand, the complexities of mitochondrial morphology in live chick heart cells. Today, we can visualise and record mitochondrial shape and dynamics with relative ease using confocal microscopy. Yet, until now, the ability to accurately and reliably define mitochondrial morphology using accessible, high throughput, automated and unbiased approaches has been largely elusive. The important studies of Cretin and co‐workers have combatted these limitations using a novel approach involving supervised machine learning algorithms, high throughput screening and automated confocal imaging, demonstrating its power by identifying novel modulators of mitochondrial morphology, as well as genetic modifiers of neurological disease underpinned by impaired mitochondrial dynamics. These robust processes certainly offer a tractable methodology that could also aid drug discovery aimed at improving mitochondrial dynamics. Currently, mitochondrial disease treatments focus on managing symptoms rather than improving biochemical function (Russell *et al,* 2020). Factors limiting the development of therapeutics include the clinical and genetic heterogeneity of mitochondrial disease, so the high throughput nature of the approach described here is particularly appealing, thus enabling a large number of drugs or small molecules to be tested across several disease cell lines in parallel.
